# Modeling Chromosomes in Mouse to Explore the Function of Genes, Genomic Disorders, and Chromosomal Organization

**DOI:** 10.1371/journal.pgen.0020086

**Published:** 2006-07-14

**Authors:** Véronique Brault, Patricia Pereira, Arnaud Duchon, Yann Hérault

**Affiliations:** University College London, United Kingdom

## Abstract

One of the challenges of genomic research after the completion of the human genome project is to assign a function to all the genes and to understand their interactions and organizations. Among the various techniques, the emergence of chromosome engineering tools with the aim to manipulate large genomic regions in the mouse model offers a powerful way to accelerate the discovery of gene functions and provides more mouse models to study normal and pathological developmental processes associated with aneuploidy. The combination of gene targeting in ES cells, recombinase technology, and other techniques makes it possible to generate new chromosomes carrying specific and defined deletions, duplications, inversions, and translocations that are accelerating functional analysis. This review presents the current status of chromosome engineering techniques and discusses the different applications as well as the implication of these new techniques in future research to better understand the function of chromosomal organization and structures.

## Introduction

Recent strategies to produce mouse lines that contain large genomic rearrangements represent a major advance to accelerate functional genomics and to provide animal models for developmental processes and human diseases such as contiguous gene and gene dosage effect syndromes. The first chromosomal rearrangements were obtained in the mouse using X-ray irradiation [1] or chemicals [2]. However, the size and position of the induced rearrangements cannot be predetermined even though the use of specific selectable markers and embryonic stem (ES) cells has improved the irradiation strategy, making it possible to generate a series of interstitial deletions at a given locus [3–6]. Homologous recombination in ES cells using replacement vectors has generated deletion of genomic fragments up to 30 kb [7,8], but inducing large defined chromosomal rearrangements was only achieved in the mid-1990s by taking advantages of the Cre/*loxP* recombinase system [9–12] and defining the chromosomal engineering strategy.

This technology led to the creation of new genetic tools for functional analysis of the mouse genome. Generation of deletions with engineered visible markers provides segmental haploidy to study recessive mutations [10,13], whereas inversions can serve as balancer chromosomes to prevent crossing-over and to facilitate large-scale mutagenesis screens for recessive lethal mutations [14,15]. The possibility of manipulating large chromosome fragments or whole chromosomes using microcell-mediated chromosome transfer (MMCT) offers the opportunity to study the function of large genes or clusters of genes and provides more and more mouse models to study human pathologies such as contiguous gene syndromes. In this review, we describe the panel of techniques available for chromosome engineering in the mouse, some of their applications for studying gene function and genomic organization and for modeling human diseases, and the implications for future research.

## Chemical and Radiation-Induced Chromosome Rearrangements

Historically, various types of rearrangements including deletions, inversions, and reciprocal translocations were obtained through irradiation or chemical mutagenesis. Such chromosomal configurations are important tools for looking at recessive lethal mutations in mice [16] or to obtain mouse models of partial aneuploidy. For example, Ts65Dn is a well-known model to study human trisomy 21 that recapitulates several phenotypic features of people with Down syndrome [17,18].

A major improvement of the radiation strategy was developed to induce a panel of deletions at a defined region [5]. Starting from the integration of a negative selection marker such as the *tk* gene into a predetermined locus by homologous recombination in a hybrid ES cell line, You et al. [5,19] were able to select for the loss of the negative marker after irradiation. The characterization of the deletions is then achieved by taking advantage of genetic markers that are polymorphic in the hybrid ES cells (Figure 1). Using this approach, Schimenti et al. [3] generated three overlapping deletion complexes spanning about 40 cM of mouse chromosome 5 (MMU5), allowing the systematic characterization of the functional regions of this chromosome by pairwise combination of the deletions through mating and serving as a model of the Wolf-Hirschhorn contiguous gene syndrome that resides in this region [3,20].

**Figure 1:**
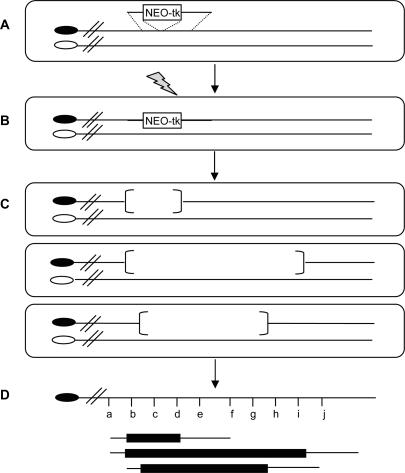
Generation and Characterization of Radiation-Induced Deletion Complexes in Mouse ES Cells (A) Insertion of a negative selectable marker (cassette Neo-tk: Hsv-thymidine kinase/neomycin resistance) into a predetermined locus by homologous recombination in F1 hybrid (129/SvJae x C57BL/6J) ES cells (C57BL/6J chromosome represented with a black centromere; 129/SvJae chromosome represented with a white centromere). (B) Treatment of the neomycin-resistant targeted cells with radiation to induce the deletions. (C) Selection in medium containing 1,2′-deoxy-2′-fluoro-β-D-arabinofuranosyl-5-iodouracil of the colonies having lost the *tk* gene. (D) Characterization of the deletion breakpoints by amplification of the DNA from these clones using primers corresponding to genetic polymorphic markers (represented under the chromosome map by letters a–j) flanking the site of the targeted integration. Deletions are represented as solid boxes.

## Using Site-Specific Recombinase to Generate Megabase Chromosome Rearrangements

### 

#### In vitro.

More defined rearrangements require the use of the Cre/*loxP* technology in mouse ES cells [9–11]. By two consecutive events one can target the *loxP* sites to predefined loci in the genome by homologous recombination. Expression of the Cre recombinase induces the desired rearrangement through site-specific recombination between the two *loxP* sites, depending on their relative position and orientation (Figure 2). For small regions (less than 100 kb), deletions and inversions can be easily generated, starting from ES cells carrying two *loxP* sites inserted in a *cis* configuration, that are treated with Cre recombinase [21,22]. However, the rearrangement occurs at a lower efficiency for larger regions. Thus, targeting vectors containing the 5′ or 3′ part of a positive selection cassette with *loxP* located downstream or upstream, respectively, were designed. Expression of the Cre recombinase results in the desired rearrangement through site-specific recombination between the two *loxP* sites, enabling the selection of the recombined allele through the restoration of the selection marker upon rearrangement. Different markers such as *Hprt* [10] or the resistance genes for neomycin [23], puromycin [24,25], or hygromycin [26] were used. As an alternative, a *tk* negative selection marker can be deleted in the rearranged locus [27–30].

**Figure 2:**
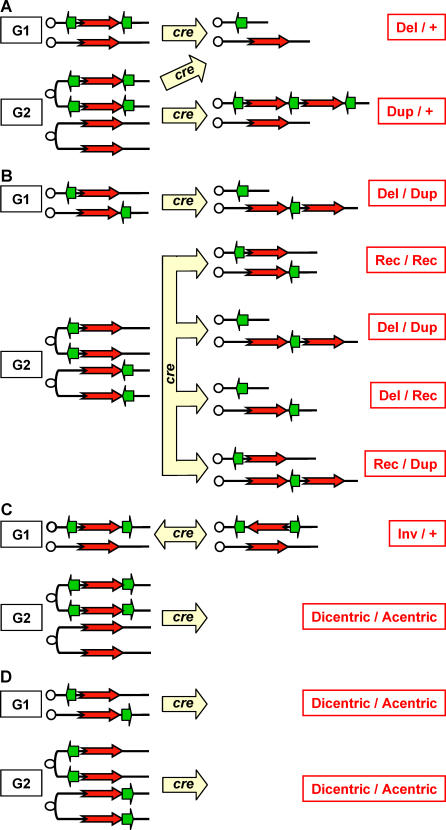
The Different Types of Chromosomal Rearrangements Produced by the Cre/*loxP* Recombinase System Deletions, duplications, or inversions can be produced depending on the relative orientation of the *loxP* sites, on their position on the homologous chromosome (i.e., in *cis* or in *trans*), and on the cell cycle stage during which the Cre-mediated recombination occurs (G1 or G2). (A) Recombination between *loxP* sites (green arrowhead) integrated in the same orientation in a *cis* configuration during the G1 phase can only generate a deletion of the region of interest (red arrow); the same configuration in the G2 phase can result in the creation of a deletion and a duplication. (B) The deletion and the corresponding duplication can also be obtained from a *trans* configuration in both G1 and G2 phases. This represents the best configuration to establish the deleted and duplicated chromosomes in the mouse, with both chromosomes compensating for each other with regard to genetic dosage, thus reducing the potential consequence of haploinsufficiency. (C and D) When the *loxP* sites are oriented in opposite directions in a *cis* configuration, an equilibrium with two forms, inverted and non-inverted, is obtained if the Cre is expressed in G1, while a more likely unstable recombined pair of acentric and dicentric chromosomes is generated if Cre reacts on *loxP* sites after the S phase or from a *trans* configuration. From all these recombinant alleles, only those containing the reconstituted mini-gene, however, will be retained during the selection in vitro. Recombinant ES-cell clones should be extensively characterized to verify the engineered chromosome (by Southern blot analysis, normal and quantitative PCR, or FISH). Del, deletion; Dup, duplication; Inv, inversion; Rec, one of the original recombinant alleles.

The efficiency of the in vitro technique, and hence its feasibility, depends on the chromosomal context and more dramatically on the design of the experiment [31–34]. Cre-mediated recombination efficiency is dependent on different factors such as the increasing distance between the *loxP* sites for a *cis* configuration (10% to 0.1% efficiency), the level of recombinase activity, and the region of interest that could induce ES-cell lethality after deletion [10,31,32,35] (unpublished data). Increasing the efficiency of the technique by a factor of 10 can be achieved by using a GFP/Cre expressing vector or classical co-transfection, followed by fluorescent-activated cell sorting of GFP+ cells that also express Cre [36,37].

Another dimension of the chromosomal engineering in vitro is the induction of mitotic recombination in G2 phase to produce selectable homozygous daughter cells from a double heterozygous mother for genetic mosaics. Such a powerful method can selectively produce ES-cell clones carrying homozygous mutations for functional recessive mutations screens in vitro, speeding up the analysis of a gene's function in cells [38,39].

Using the in vitro technique requires two targeting vectors for each bordering loci that could contain different types of selectable cassettes for inducing and selecting chromosomal rearrangements in various types of ES cells. But in the case of the restoration of the HPRT function, the use of *Hprt*-deficient ES cells such as AB2.2 [10] or HM-1 [40] is required. Nevertheless, this is a method of choice given the number of ready-to-use targeting vectors for the *Hprt* selection system available from the Mutagenic Insertion and Chromosome Engineering Resource [41] (MICER; http://www.sanger.ac.uk/micer/), the use of retroviral vectors to target the second *loxP* site [42–44], and the panel of rearranged chromosomes that has been engineered [15,41]. Furthermore, the presence of coat color markers in those targeting vectors allows an easy discrimination of mice carrying the recombined chromosome [13].

The in vitro strategy was developed extensively to study specific loci containing large genes or clusters of genes (see Table 1) [10,21,22,25–28,45–56], to generate translocations or deletions similar to those found in cancer [9,11,30–32,57–62], or to create models of contiguous gene syndromes, such as the Smith-Magenis [44,63–66], Prader-Willy [67], and DiGeorge syndromes [43,68,69]. Similarly, new mouse models of Down syndrome have been generated to study the impact of a critical region identified in human Chromosome 21 [23]. Taken together, these data illustrate how large-scale chromosomal engineering is a powerful tool to unravel genes with dosage effects and their contribution to aneuploid syndromes in the mouse model.

**Table 1:**
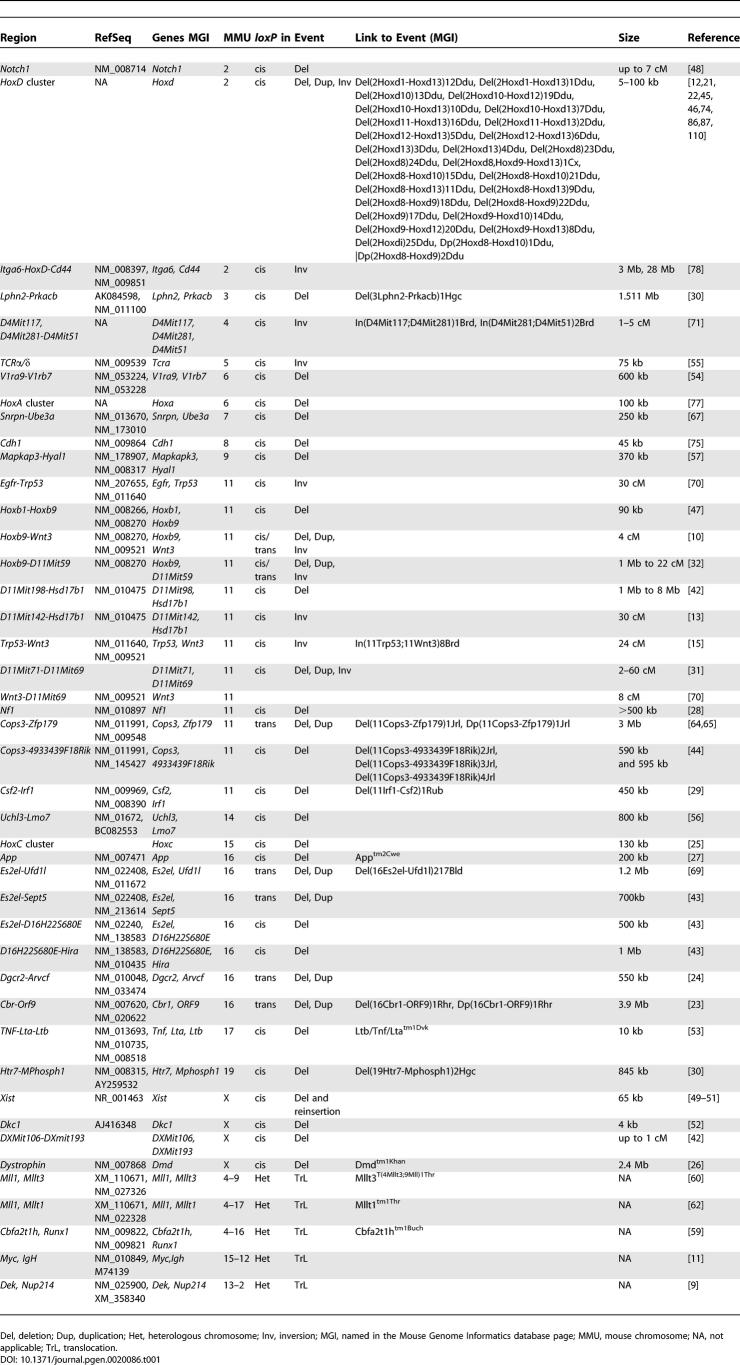
Genomic Rearrangements Induced in the Mouse Genome by Chromosome Engineering

Unraveling lethal mutations is difficult in classical mutagenesis screens, unless a chromosome carrying an inversion is used as a balancer chromosome [15]. The production of inversions by chromosome engineering in the mouse offers the advantage of controlling the size of the inverted region and of generating inversions that will induce lethality in homozygous state, such as those obtained on mouse Chromosome 11 that disrupt the *Wnt3* gene [14,31,32,70] or on Chromosome 4 [71]. Using such a balancer chromosome approach associated with chemical mutagenesis, two mutagenesis screens led to the characterization of, respectively, 59 recessive lethal mutations on mouse Chromosome 11, and 19 mutations on mouse Chromosome 4 [14,72].

#### In vivo.

Cre-mediated recombination is widely used to generate conditional mutagenesis for a gene of interest in a tissue- or time-dependent manner [73]. Similarly, several groups succeeded in generating in vivo large deletions, duplications, inversions, and translocations (Table 1). This can be carried out between two *loxP* sites in *cis* either inserted in ES cells [22,74–76] or selected after classical crossing-over between two original founder lines by the sequential targeted recombination induced genomic rearrangement (STRING) (Figure 3), [24,77,78]. Furthermore, the targeted meiotic recombination (TAMERE) strategy allows to obtain both a deletion and a duplication of a region of up to 150 kb, starting from two *loxP* sites, in a *trans* configuration, with a frequency that can vary from 1% to 10% (Figure 3) [12,79]. Similarly to in vitro, the recombination frequency decreases in vivo for large regions. Nevertheless it is still effective for *cis* configuration (0.3%–1%) separated by up to 28 Mb [78], but seems to be not workable with *loxP* in *trans* distant from 3.9 Mb [34]. Thus, the STRING approach [78] offers an interesting alternative to the MICER strategy, without the need for extensive technological investments, but still requires an efficient and large genotyping program.

**Figure 3:**
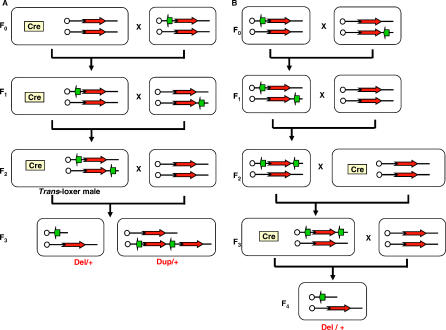
Strategies for In Vivo Cre-Mediated Recombination (A) The general principle of TAMERE is based on two successive breedings in order to have in one male, named the trans-loxer, the *Sycp1Cre* (*Synaptonemal Complex protein 1*) transgene and the two *loxP* sites in a *trans* configuration, inserted previously in the same orientation at each targeted locus, that define the genetic interval. The *Synaptonemal Complex protein 1* promoter drives Cre expression at prophase of meiosis in male spermatocytes when chromatid pairs are closely aligned, in order to facilitate the chromatid exchange, leading to the formation of the deletion and the duplication of the interval delimited by the two *loxP* sites. The last step consists in mating trans-loxer males with wild-type females to generate, in the progeny, individuals carrying the deletion or the duplication of the targeted region. (B) The STRING approach takes advantage of a classical crossing-over to bring the two *loxP* sites into a *cis* configuration to generate a deletion. Two parental mice (F_0_) carrying *loxP* sites flanking a selected region are crossed. The F_1_ progeny containing the two *loxP* sites are then mated to wild-type mice. The offspring are screened for meiotic crossing-over between both sites leading to mice carrying the *loxP* sites in a *cis* configuration. In the subsequent cross, a ubiquitously expressed Cre transgene is introduced, generating the deletion that is established in the next F_4_ generation. Del, deletion; Dup, duplication. red arrow, region of interest; green arrow, *loxP* site.

In vivo chromosomal engineering during mitosis often leads to mosaic individuals in the first progeny in which the *Cre* transgene is combined with *loxP* sites in *cis* [74,80]. The level of mosaicism found depends on a balance between the size of the targeted region and Cre activity. For example, Mersher et al. (2001) noticed 17% of mosaic mice for a 1.5-Mb deletion using the *ZP3-Cre* line expressed in the oocytes, and we found 26% of mice showing a mosaic profile for a 0.7-Mb induced deletion using a *CMV-Cre* expressing line [81]. Genetic mosaicism could be an advantage for lineage analysis as Cre-induced rearrangements can be used to label and trace a cell population in vivo [82]. Thus, it is crucial to well characterize the Cre transgenic lines, by analyzing the expression of Cre mRNA, or the Cre activity by mating with a mouse carrying a reporter gene such as the R26R [83] and ACZL lines [84].

Large rearrangements can lead to early embryonic lethality. Hence, absence of mice carrying the new engineered chromosome in the in vivo approach could be caused by a failure of Cre-mediated recombination but could also depend on the lethal effect of the new genetic configuration [85]. To address such questions, it is important to test for the presence of the new chromosomal configuration in the carrier double-transgenic animal [78]. An alternative would be to compensate the deletion with a balanced duplication [85]. Unfortunately, an efficient strategy needs to be explored to induce such *trans* recombination in vivo for large regions. An additional way to overcome this problem is to trigger the recombination in a time- and/or tissue-specific manner, but only a few reports so far have described this option [31,61,62].

In vivo approaches to making deletions and inversions were largely used to explore the function and regulation of genes (Table 1). The best example for such studies is the extensive work done over the last ten years by D. Duboule and collaborators to analyze the molecular mechanisms that modulate expression of *Hox* genes encoding transcription factors controlling positional information along the trunk and limb axes [22,45,46,76,86–88]. In particular, they have combined the in vivo strategy with knock-in of a *lacZ* reporter gene, to trace the consequences of the induced rearrangements on the expression of the *Hoxd* genes during embryonic development. Similar deletion approaches were used to study *cadherin 1* gene regulation [75]. Herault et al. [89] extended this strategy by replacing the *LacZ* by the *Cre* to provide a tool to specifically inactivate *Hox* genes in progressively more extended domains of *Hox* expression. On the whole, the in vivo strategy appears very attractive as it is less expensive, doesn't need the settling of a large breeding program, and avoids a few complicated steps of cell culturing thanks to the increasing availability of mouse or ES cell lines containing *loxP* sites (Table 2).

**Table 2:**
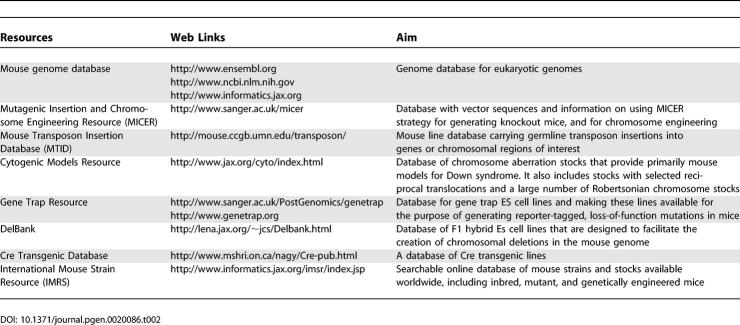
Resources Available Online for Chromosomal Engineering in Mice

The mosaic analysis with double markers [82] combines chimeric fluorescent constructs and G2-induced mitotic recombination in vivo. Such a strategy allows determination of the consequence of mutation by visualization of double-colored mutated cells, enabling high-resolution lineage tracing in vivo to elucidate biological processes, such as cell fate in the nervous system. The development of such a strategy will lead to genetic mosaic analysis, bypassing the problem of the lethality linked to some mutations by exploring the mosaic condition.

## Transchromosomal Lines

The Cre/*loxP* system allows the modeling of native chromosomes while MMCT enables manipulation of large fragments or whole chromosomes from various species and particularly human with the making of transchromosomic lines. This technique originally developed in the 1970s, is based on the fusion of microcells, containing single or small numbers of chromosomes, with whole cells in order to transfer exogenous chromosome material into host cells (for a review, see [90,91]). MMCT can be carried out with somatic cells, embryonic carcinoma cells or ES cells as recipients. Applications of MMCT are numerous, ranging from hunting for tumor suppressor, DNA repair or senescence-inducing genes, assessing genomic instability, imprinting and chromatin modification, constructing and manipulating artificial chromosomes for potential gene therapies to act as vectors for specific genes or genomic regions, and expressing proteins or studying aspects of chromosome behaviour in mitosis and meiosis [92–95].

A further application is the creation of mouse lines that carry fragments or whole human chromosomes as freely segregating extra chromosomes. To achieve this, transchromosomic mouse ES cells were injected into mouse blastocysts to produce mouse chimeras. The first transchromosomal animals were produced in the late 1990s and carried different human chromosome fragments as freely segregating extra chromosomes [96]. Using this technique, models of Down syndrome were obtained in chimeric mice with transchromosomal ES cells containing different parts of human Chromosome 21 (Hsa21), ranging from ~50 to ~0.2 Mb [97.98]. But the chromosome fragments tended to be lost during development, leading to phenotypic variations. In spite of this, the chimeric animals exhibited behavioral impairments and cardiac defects similar to those described in humans with Down syndrome [99,100]. E. Fisher and collaborators succeeded in producing a trans-species aneuploid mouse line, called Tc1, that stably transmitted almost a complete Hsa21, generating a more full model of Down syndrome [101]. Indeed, the Tc1 line displays a large set of deficits similar to those observed in trisomic 21 patients that were not found in earlier models. The previously observed failure of transchromosomal germline transmission was overcame by using female mouse ES cell lines to derive female chimeras that support the transmission of the aneuploid chromosome through the germline [97]. Nevertheless such transchromosomic lines are difficult to obtain and to work with. A large number of ES cell clones should be controlled to detect any rearrangements and should be injected to recover a germline transmission. In addition, transmission from Tc1 females only could be achieved with a rate of 40% in a hybrid genetic background, and a large panel of animals should be studied as the aneuploid chromosome tends to be lost during mitosis with variable rates [101]. Nonetheless, transchromosomic lines offer a promising substitute to mouse segmental aneuploidies, as the complete genomic sequence is included, and hence modeling a more complete human aneuploidy.

## Conclusion

Chromosome engineering combined with the transchromosomic approach is widely used for dissecting the function, the regulation, and the contribution of genes to genetic disorders, such as contiguous gene and aneuploidy syndromes. For example, the increasing number of mouse models for trisomy 21 that have been created recently [101,102] provides the necessary tools to understand how dosage imbalance results in the abnormal phenotypes observed in the human patients. Breeding of the different mouse strains carrying either the duplication or the deletion of human Chromosome 21 syntenic regions will not only allow the creation of a full model for human trisomy 21, but also the deciphering of genetic interaction between regions and enable one to look for candidate genes for each phenotype. This approach is also valid for evaluating the effect of copy number variation of genomic regions observed in the human population that might contribute to the susceptibility to certain diseases [103–105]. Manipulating chromosomes on a large scale is likely to be increasingly developed in the future also to identify genes underlying complex phenotypes of polygenic diseases such as diabetes, cancer, asthma, or obesity caused by a combination of environmental and multiple genetic factors. To this end, transchromosomic lines combined with deletions might be a promising way to obtain more humanized models to study specific human genes and pathologies.

Even though both techniques still require considerable effort, they benefit from the development of the MICER resource, with more than 18,000 targeting vectors referenced in the mouse genome database, the increasing number of mouse carrying *loxP* sites at various loci and of Cre transgenic lines (Table 2). Together with the in vivo approaches TAMERE and STRING [12,78], any laboratory can now manipulate large genomic regions without any additional work on ES cells, in order to analyze further the genomic organization or to derive new tools for genetic analysis (deletion, duplication, and inversion). These different technologies are further supported by the combined effort of the scientific community to establish large-scale gene trap mutagenesis programs, integrating Cre/*loxP* technology and offering the possibility to create a variety of alleles [106–108].

Emerging from these strategies of chromosome engineering is the fascinating aspect of chromosome organization, structure, and function, implicated in the as yet poorly understood code for genetic instructions. Indeed, the human genome contains about 2% of coding sequence, RNA genes, and regulatory regions. In some cases, control regions lay at a great distance from the gene, and hence the long-range interaction is only detected by manipulation of large chromosomal regions [109–112]. Surprisingly, a significant amount of the non-coding portion of the genome is under active selection, suggesting that it is also functionally important, yet little is known about it [113–116]. Large desert gene regions also are found and are starting to be investigated by using chromosome engineering [30]. We speculate that the modeling of chromosome will be more commonly used to better understand the role of non-coding sequences, and may be used to decipher the “C-value enigma” [117] and the chromosome architecture lying at the heart of genetic instructions. Our increased ability to manipulate the mammalian genome provides us with new tools for the development of a functional chromoso–genomic approach, bringing an exciting new dimension into biological and biomedical research.

## Supporting Information

### Accession Numbers

The accession numbers of genes mentioned in this paper from the GenBank database (http://www.ncbi.nlm.nih.gov/entrez) are *Wnt3* gene (NM_009521.1) and *cadherin 1* (NM_009864.1).

## Abbreviations

ES: embryonic stem
MMCT: microcell-mediated chromosome transfer
STRING: sequential targeted recombination induced genomic rearrangement
TAMERE: targeted meiotic recombination

## References

[pgen-0020086-b001] Green EL, Roderick TH, Green EL (1966). Radiation genetics. Biology of the laboratory mouse.

[pgen-0020086-b002] Russell LB, Hunsicker PR, Cacheiro NL, Bangham JW, Russell WL (1989). Chlorambucil effectively induces deletion mutations in mouse germ cells. Proc Natl Acad Sci U S A.

[pgen-0020086-b003] Schimenti JC, Libby BJ, Bergstrom RA, Wilson LA, Naf D (2000). Interdigitated deletion complexes on mouse chromosome 5 induced by irradiation of embryonic stem cells. Genome Res.

[pgen-0020086-b004] Goodwin NC, Ishida Y, Hartford S, Wnek C, Bergstrom RA (2001). DelBank: A mouse ES-cell resource for generating deletions. Nat Genet.

[pgen-0020086-b005] You Y, Bergstrom R, Klemm M, Lederman B, Nelson H (1997). Chromosomal deletion complexes in mice by radiation of embryonic stem cells. Nat Genet.

[pgen-0020086-b006] Kushi A, Edamura K, Noguchi M, Akiyama K, Nishi Y (1998). Generation of mutant mice with large chromosomal deletion by use of irradiated ES cells—Analysis of large deletion around hprt locus of ES cell. Mamm Genome.

[pgen-0020086-b007] Zhang H, Hasty P, Bradley A (1994). Targeting frequency for deletion vectors in embryonic stem cells. Mol Cell Biol.

[pgen-0020086-b008] Gu H, Zou YR, Rajewsky K (1993). Independent control of immunoglobulin switch recombination at individual switch regions evidenced through Cre-loxP-mediated gene targeting. Cell.

[pgen-0020086-b009] Van Deursen J, Fornerod M, Van Rees B, Grosveld G (1995). Cre-mediated site-specific translocation between nonhomologous mouse chromosomes. Proc Natl Acad Sci U S A.

[pgen-0020086-b010] Ramirez-Solis R, Liu P, Bradley A (1995). Chromosome engineering in mice. Nature.

[pgen-0020086-b011] Smith AJ, De Sousa MA, Kwabi-Addo B, Heppell-Parton A, Impey H (1995). A site-directed chromosomal translocation induced in embryonic stem cells by Cre-loxP recombination. Nat Genet.

[pgen-0020086-b012] Herault Y, Rassoulzadegan M, Cuzin F, Duboule D (1998). Engineering chromosomes in mice through targeted meiotic recombination (TAMERE). Nat Genet.

[pgen-0020086-b013] Zheng B, Mills AA, Bradley A (1999). A system for rapid generation of coat color-tagged knockouts and defined chromosomal rearrangements in mice. Nucleic Acids Res.

[pgen-0020086-b014] Kile BT, Hentges KE, Clark AT, Nakamura H, Salinger AP (2003). Functional genetic analysis of mouse chromosome 11. Nature.

[pgen-0020086-b015] Zheng B, Sage M, Cai WW, Thompson DM, Tavsanli BC (1999). Engineering a mouse balancer chromosome. Nat Genet.

[pgen-0020086-b016] Wilson L, Ching YH, Farias M, Hartford SA, Howell G (2005). Random mutagenesis of proximal mouse chromosome 5 uncovers predominantly embryonic lethal mutations. Genome Res.

[pgen-0020086-b017] Davisson MT, Schmidt C, Akeson EC (1990). Segmental trisomy of murine chromosome 16: A new model system for studying Down syndrome. Prog Clin Biol Res.

[pgen-0020086-b018] Reeves RH, Irving NG, Moran TH, Wohn A, Kitt C (1995). A mouse model for Down syndrome exhibits learning and behaviour deficits. Nat Genet.

[pgen-0020086-b019] You Y, Bersgtram R, Klemm M, Nelson H, Jaenisch R (1998). Utility of C57BL/6J × 129/SvJae embryonic stem cells for generating chromosomal deletions: Tolerance to gamma radiation and microsatellite polymorphism. Mamm Genome.

[pgen-0020086-b020] Naf D, Wilson LA, Bergstrom RA, Smith RS, Goodwin NC (2001). Mouse models for the Wolf-Hirschhorn deletion syndrome. Hum Mol Genet.

[pgen-0020086-b021] Spitz F, Gonzalez F, Peichel C, Vogt TF, Duboule D (2001). Large scale transgenic and cluster deletion analysis of the HoxD complex separate an ancestral regulatory module from evolutionary innovations. Genes Dev.

[pgen-0020086-b022] Zakany J, Duboule D (1996). Synpolydactyly in mice with a targeted deficiency in the HoxD complex. Nature.

[pgen-0020086-b023] Olson LE, Richtsmeier JT, Leszl J, Reeves RH (2004). A chromosome 21 critical region does not cause specific Down syndrome phenotypes. Science.

[pgen-0020086-b024] Puech A, Saint-Jore B, Merscher S, Russell RG, Cherif D (2000). Normal cardiovascular development in mice deficient for 16 genes in 550 kb of the velocardiofacial/DiGeorge syndrome region. Proc Natl Acad Sci U S A.

[pgen-0020086-b025] Suemori H, Noguchi S (2000). Hox C cluster genes are dispensable for overall body plan of mouse embryonic development. Dev Biol.

[pgen-0020086-b026] Kudoh H, Ikeda H, Kakitani M, Ueda A, Hayasaka M (2005). A new model mouse for Duchenne muscular dystrophy produced by 2.4 Mb deletion of dystrophin gene using Cre-loxP recombination system. Biochem Biophys Res Commun.

[pgen-0020086-b027] Li ZW, Stark G, Gotz J, Rulicke T, Gschwind M (1996). Generation of mice with a 200-kb amyloid precursor protein gene deletion by Cre recombinase-mediated site-specific recombination in embryonic stem cells. Proc Natl Acad Sci U S A.

[pgen-0020086-b028] Schlake T, Schupp I, Kutsche K, Mincheva A, Lichter P (1999). Predetermined chromosomal deletion encompassing the Nf-1 gene. Oncogene.

[pgen-0020086-b029] Zhu Y, Jong MC, Frazer KA, Gong E, Krauss RM (2000). Genomic interval engineering of mice identifies a novel modulator of triglyceride production. Proc Natl Acad Sci U S A.

[pgen-0020086-b030] Nobrega MA, Zhu Y, Plajzer-Frick I, Afzal V, Rubin EM (2004). Megabase deletions of gene deserts result in viable mice. Nature.

[pgen-0020086-b031] Zheng B, Sage M, Sheppeard EA, Jurecic V, Bradley A (2000). Engineering mouse chromosomes with Cre-loxP: Range, efficiency, and somatic applications. Mol Cell Biol.

[pgen-0020086-b032] Liu P, Zhang H, McLellan A, Vogel H, Bradley A (1998). Embryonic lethality and tumorigenesis caused by segmental aneuploidy on mouse chromosome 11. Genetics.

[pgen-0020086-b033] Yu Y, Bradley A (2001). Engineering chromosomal rearrangements in mice. Nat Rev Genet.

[pgen-0020086-b034] Olson LE, Tien J, South S, Reeves RH (2005). Long-range chromosomal engineering is more efficient in vitro than in vivo. Transgenic Res.

[pgen-0020086-b035] Baubonis W, Sauer B (1993). Genomic targeting with purified Cre recombinase. Nucleic Acids Res.

[pgen-0020086-b036] Soukharev S, Miller JL, Sauer B (1999). Segmental genomic replacement in embryonic stem cells by double lox targeting. Nucleic Acids Res.

[pgen-0020086-b037] Gagneten S, Le Y, Miller J, Sauer B (1997). Brief expression of a GFP cre fusion gene in embryonic stem cells allows rapid retrieval of site-specific genomic deletions. Nucleic Acids Res.

[pgen-0020086-b038] Liu P, Jenkins NA, Copeland NG (2002). Efficient Cre-loxP-induced mitotic recombination in mouse embryonic stem cells. Nat Genet.

[pgen-0020086-b039] Koike H, Horie K, Fukuyama H, Kondoh G, Nagata S (2002). Efficient biallelic mutagenesis with Cre/loxP-mediated inter-chromosomal recombination. EMBO Rep.

[pgen-0020086-b040] Magin TM, McWhir J, Melton DW (1992). A new mouse embryonic stem cell line with good germ line contribution and gene targeting frequency. Nucleic Acids Res.

[pgen-0020086-b041] Adams DJ, Biggs PJ, Cox T, Davies R, van der Weyden L (2004). Mutagenic insertion and chromosome engineering resource (MICER). Nat Genet.

[pgen-0020086-b042] Su H, Wang X, Bradley A (2000). Nested chromosomal deletions induced with retroviral vectors in mice. Nat Genet.

[pgen-0020086-b043] Lindsay EA, Vitelli F, Su H, Morishima M, Huynh T (2001). Tbx1 haploinsufficieny in the DiGeorge syndrome region causes aortic arch defects in mice. Nature.

[pgen-0020086-b044] Yan J, Keener VW, Bi W, Walz K, Bradley A (2004). Reduced penetrance of craniofacial anomalies as a function of deletion size and genetic background in a chromosome engineered partial mouse model for Smith-Magenis syndrome. Hum Mol Genet.

[pgen-0020086-b045] Kondo T, Zakany J, Duboule D (1998). Control of colinearity in AbdB genes of the mouse HoxD complex. Mol Cell.

[pgen-0020086-b046] Kondo T, Duboule D (1999). Breaking colinearity in the mouse HoxD complex. Cell.

[pgen-0020086-b047] Medina-Martinez O, Bradley A, Ramirez-Solis R (2000). A large targeted deletion of Hoxb1-Hoxb9 produces a series of single-segment anterior homeotic transformations. Dev Biol.

[pgen-0020086-b048] LePage DF, Church DM, Millie E, Hassold TJ, Conlon RA (2000). Rapid generation of nested chromosomal deletions on mouse chromosome 2. Proc Natl Acad Sci U S A.

[pgen-0020086-b049] Clerc P, Avner P (1998). Role of the region 3′ to Xist exon 6 in the counting process of X-chromosome inactivation. Nat Genet.

[pgen-0020086-b050] Morey C, Arnaud D, Avner P, Clerc P (2001). Tsix-mediated repression of Xist accumulation is not sufficient for normal random X inactivation. Hum Mol Genet.

[pgen-0020086-b051] Morey C, Navarro P, Debrand E, Avner P, Rougeulle C (2004). The region 3′ to Xist mediates X chromosome counting and H3 Lys-4 dimethylation within the Xist gene. EMJO J.

[pgen-0020086-b052] He J, Navarrete S, Jasinski M, Vulliamy T, Dokal I (2002). Targeted disruption of Dkc1, the gene mutated in X-linked dyskeratosis congenita, causes embryonic lethality in mice. Oncogene.

[pgen-0020086-b053] Kuprash DV, Alimzhanov MB, Tumanov AV, Grivennikov SI, Shakhov AN (2002). Redundancy in tumor necrosis factor (TNF) and lymphotoxin (LT) signaling in vivo: Mice with inactivation of the entire TNF/LT locus versus single-knockout mice. Mol Cell Biol.

[pgen-0020086-b054] Del Punta K, Leinders-Zufall T, Rodriguez I, Jukam D, Wysocki CJ (2002). Deficient pheromone responses in mice lacking a cluster of vomeronasal receptor genes. Nature.

[pgen-0020086-b055] Khor B, Wehrly TD, Sleckman BP (2005). Chromosomal excision of TCRdelta chain genes is dispensable for alphabeta T cell lineage commitment. Int Immunol.

[pgen-0020086-b056] Semenova E, Wang X, Jablonski MM, Levorse J, Tilghman SM (2003). An engineered 800 kilobase deletion of Uchl3 and Lmo7 on mouse chromosome 14 causes defects in viability, postnatal growth and degeneration of muscle and retina. Hum Mol Genet.

[pgen-0020086-b057] Smith AJ, Xian J, Richardson M, Johnstone KA, Rabbitts PH (2002). Cre-loxP chromosome engineering of a targeted deletion in the mouse corresponding to the 3p21.3 region of homozygous loss in human tumours. Oncogene.

[pgen-0020086-b058] Biggs PJ, Vogel H, Sage M, Martin LA, Donehower LA (2003). Allelic phasing of a mouse chromosome 11 deficiency influences p53 tumorigenicity. Oncogene.

[pgen-0020086-b059] Buchholz F, Refaeli Y, Trumpp A, Bishop JM (2000). Inducible chromosomal translocation of AML1 and ETO genes through Cre/loxP-mediated recombination in the mouse. EMBO Rep.

[pgen-0020086-b060] Collins EC, Pannell R, Simpson EM, Forster A, Rabbitts TH (2000). Inter-chromosomal recombination of Mll and Af9 genes mediated by cre-loxP in mouse development. EMBO Rep.

[pgen-0020086-b061] Drynan LF, Pannell R, Forster A, Chan NM, Cano F (2005). Mll fusions generated by Cre-loxP-mediated de novo translocations can induce lineage reassignment in tumorigenesis. EMBO J.

[pgen-0020086-b062] Forster A, Pannell R, Drynan LF, McCormack M, Collins EC (2003). Engineering de novo reciprocal chromosomal translocations associated with Mll to replicate primary events of human cancer. Cancer Cell.

[pgen-0020086-b063] Bi W, Ohyama T, Nakamura H, Yan J, Visvanathan J (2005). Inactivation of Rai1 in mice recapitulates phenotypes observed in chromosome engineered mouse models for Smith-Magenis syndrome. Hum Mol Genet.

[pgen-0020086-b064] Walz K, Caratini-Rivera S, Bi W, Fonseca P, Mansouri DL (2003). Modeling del(17)(p11.2p11.2) and dup(17)(p11.2p11.2) contiguous gene syndromes by chromosome engineering in mice: Phenotypic consequences of gene dosage imbalance. Mol Cell Biol.

[pgen-0020086-b065] Walz K, Spencer C, Kaasik K, Lee CC, Lupski JR (2004). Behavioral characterization of mouse models for Smith-Magenis syndrome and dup(17)(p11.2p11.2). Hum Mol Genet.

[pgen-0020086-b066] Lupski JR, Stankiewicz P (2005). Genomic disorders: Molecular mechanisms for rearrangements and conveyed phenotypes. PLoS Genet.

[pgen-0020086-b067] Tsai TF, Jiang YH, Bressler J, Armstrong D, Beaudet AL (1999). Paternal deletion from Snrpn to Ube3a in the mouse causes hypotonia, growth retardation and partial lethality and provides evidence for a gene contributing to Prader-Willi syndrome. Hum Mol Genet.

[pgen-0020086-b068] Scambler PJ (2000). The 22q11 deletion syndromes. Hum Mol Genet.

[pgen-0020086-b069] Lindsay EA, Botta A, Jurecic V, Carattini-Rivera S, Cheah YC (1999). Congenital heart disease in mice deficient for the DiGeorge syndrome region. Nature.

[pgen-0020086-b070] Klysik J, Dinh C, Bradley A (2004). Two new mouse chromosome 11 balancers. Genomics.

[pgen-0020086-b071] Nishijima I, Mills A, Qi Y, Mills M, Bradley A (2003). Two new balancer chromosomes on mouse chromosome 4 to facilitate functional annotation of human chromosome 1p. Genesis.

[pgen-0020086-b072] Hentges KE, Nakamura H, Furuta Y, Yu Y, Thompson DM (2006). Novel lethal mouse mutants produced in balancer chromosome screens. Gene Expr Patterns.

[pgen-0020086-b073] Gu H, Marth JD, Orban PC, Mossmann H, Rajewsky K (1994). Deletion of a DNA polymerase beta gene segment in T cells using cell type-specific gene targeting. Science.

[pgen-0020086-b074] Zakany J, Kmita M, Duboule D (2004). A dual role for Hox genes in limb anterior-posterior asymmetry. Science.

[pgen-0020086-b075] Stemmler MP, Hecht A, Kemler R (2005). E-cadherin intron 2 contains cis-regulatory elements essential for gene expression. Development.

[pgen-0020086-b076] Kmita M, van Der Hoeven F, Zakany J, Krumlauf R, Duboule D (2000). Mechanisms of Hox gene colinearity: Transposition of the anterior Hoxb1 gene into the posterior HoxD complex. Genes Dev.

[pgen-0020086-b077] Kmita M, Tarchini B, Zakany J, Logan M, Tabin CJ (2005). Early developmental arrest of mammalian limbs lacking HoxA/HoxD gene function. Nature.

[pgen-0020086-b078] Spitz F, Herkenne C, Morris MA, Duboule D (2005). Inversion-induced disruption of the Hoxd cluster leads to the partition of regulatory landscapes. Nat Genet.

[pgen-0020086-b079] Genoud N, Behrens A, Miele G, Robay D, Heppner FL (2004). Disruption of Doppel prevents neurodegeneration in mice with extensive Prnp deletions. Proc Natl Acad Sci U S A.

[pgen-0020086-b080] Leneuve P, Colnot S, Hamard G, Francis F, Niwa-Kawakita M (2003). Cre-mediated germline mosaicism: A new transgenic mouse for the selective removal of residual markers from tri-lox conditional alleles. Nucleic Acids Res.

[pgen-0020086-b081] Dupe V, Davenne M, Brocard J, Dolle P, Mark M (1997). In vivo functional analysis of the Hoxa-1 3′ retinoic acid response element (3′RARE). Development.

[pgen-0020086-b082] Zong H, Espinosa JS, Su HH, Muzumdar MD, Luo L (2005). Mosaic analysis with double markers in mice. Cell.

[pgen-0020086-b083] Soriano P (1999). Generalized lacZ expression with the ROSA26 Cre reporter strain. Nat Genet.

[pgen-0020086-b084] Akagi K, Sandig V, Vooijs M, Van der Valk M, Giovannini M (1997). Cre-mediated somatic site-specific recombination in mice. Nucleic Acids Res.

[pgen-0020086-b085] Yu YE, Morishima M, Pao A, Wang DY, Wen XY (2006). A deficiency in the region homologous to human 17q21.33-q23.2 causes heart defects in mice. Genetics.

[pgen-0020086-b086] Kmita M, Fraudeau N, Herault Y, Duboule D (2002). Serial deletions and duplications suggest a mechanism for the collinearity of Hoxd genes in limbs. Nature.

[pgen-0020086-b087] Kmita M, Kondo T, Duboule D (2000). Targeted inversion of a polar silencer within the HoxD complex re-allocates domains of enhancer sharing. Nat Genet.

[pgen-0020086-b088] Kmita M, Tarchini B, Duboule D, Herault Y (2002). Evolutionary conserved sequences are required for the insulation of the vertebrate Hoxd complex in neural cells. Development.

[pgen-0020086-b089] Herault Y, Kmita M, Sawaya CC, Duboule D (2002). A nested deletion approach to generate Cre deleter mice with progressive Hox profiles. Int J Dev Biol.

[pgen-0020086-b090] Doherty AM, Fisher EM (2003). Microcell-mediated chromosome transfer (MMCT): Small cells with huge potential. Mamm Genome.

[pgen-0020086-b091] Meaburn KJ, Parris CN, Bridger JM (2005). The manipulation of chromosomes by mankind: The uses of microcell-mediated chromosome transfer. Chromosoma.

[pgen-0020086-b092] Kuroiwa Y, Tomizuka K, Shinohara T, Kazuki Y, Yoshida H (2000). Manipulation of human minichromosomes to carry greater than megabase-sized chromosome inserts. Nat Biotechnol.

[pgen-0020086-b093] Voet T, Liebe B, Labaere C, Marynen P, Scherthan H (2003). Telomere-independent homologue pairing and checkpoint escape of accessory ring chromosomes in male mouse meiosis. J Cell Biol.

[pgen-0020086-b094] Shen MH, Mee PJ, Nichols J, Yang J, Brook F (2000). A structurally defined mini-chromosome vector for the mouse germ line. Curr Biol.

[pgen-0020086-b095] Kakeda M, Hiratsuka M, Nagata K, Kuroiwa Y, Kakitani M (2005). Human artificial chromosome (HAC) vector provides long-term therapeutic transgene expression in normal human primary fibroblasts. Gene Ther.

[pgen-0020086-b096] Tomizuka K, Yoshida H, Uejima H, Kugoh H, Sato K (1997). Functional expression and germline transmission of a human chromosome fragment in chimaeric mice. Nat Genet.

[pgen-0020086-b097] Hernandez D, Mee PJ, Martin JE, Tybulewicz VL, Fisher EM (1999). Transchromosomal mouse embryonic stem cell lines and chimeric mice that contain freely segregating segments of human chromosome 21. Hum Mol Genet.

[pgen-0020086-b098] Shinohara T, Tomizuka K, Takehara S, Yamauchi K, Katoh M (2000). Stability of transferred human chromosome fragments in cultured cells and in mice. Chromosome Res.

[pgen-0020086-b099] Shinohara T, Tomizuka K, Miyabara S, Takehara S, Kazuki Y (2001). Mice containing a human chromosome 21 model behavioral impairment and cardiac anomalies of Down's syndrome. Hum Mol Genet.

[pgen-0020086-b100] Kazuki Y, Shinohara T, Tomizuka K, Katoh M, Ohguma A (2001). Germline transmission of a transferred human chromosome 21 fragment in transchromosomal mice. J Hum Genet.

[pgen-0020086-b101] O'Doherty A, Ruf S, Mulligan C, Hildreth V, Errington ML (2005). An aneuploid mouse strain carrying human chromosome 21 with Down syndrome phenotypes. Science.

[pgen-0020086-b102] Olson LE, Roper RJ, Baxter LL, Carlson EJ, Epstein CJ (2004). Down syndrome mouse models Ts65Dn, Ts1Cje, and Ms1Cje/Ts65Dn exhibit variable severity of cerebellar phenotypes. Dev Dyn.

[pgen-0020086-b103] Goidts V, Armengol L, Schempp W, Conroy J, Nowak N (2006). Identification of large-scale human-specific copy number differences by inter-species array comparative genomic hybridization.

[pgen-0020086-b104] Aitman TJ, Dong R, Vyse TJ, Norsworthy PJ, Johnson MD (2006). Copy number polymorphism in Fcgr3 predisposes to glomerulonephritis in rats and humans. Nature.

[pgen-0020086-b105] Sharp AJ, Locke DP, McGrath SD, Cheng Z, Bailey JA (2005). Segmental duplications and copy-number variation in the human genome. Am J Hum Genet.

[pgen-0020086-b106] Austin CP, Battey JF, Bradley A, Bucan M, Capecchi M (2004). The knockout mouse project. Nat Genet.

[pgen-0020086-b107] Auwerx J, Avner P, Baldock R, Ballabio A, Balling R (2004). The European dimension for the mouse genome mutagenesis program. Nat Genet.

[pgen-0020086-b108] Glaser S, Anastassiadis K, Stewart AF (2005). Current issues in mouse genome engineering. Nat Genet.

[pgen-0020086-b109] Sagai T, Hosoya M, Mizushina Y, Tamura M, Shiroishi T (2005). Elimination of a long-range cis-regulatory module causes complete loss of limb-specific Shh expression and truncation of the mouse limb. Development.

[pgen-0020086-b110] Tarchini B, Duboule D (2006). Control of Hoxd genes' collinearity during early limb development. Dev Cell.

[pgen-0020086-b111] Patrinos GP, de Krom M, de Boer E, Langeveld A, Imam AM (2004). Multiple interactions between regulatory regions are required to stabilize an active chromatin hub. Genes Dev.

[pgen-0020086-b112] Hadchouel J, Carvajal JJ, Daubas P, Bajard L, Chang T (2003). Analysis of a key regulatory region upstream of the Myf5 gene reveals multiple phases of myogenesis, orchestrated at each site by a combination of elements dispersed throughout the locus. Development.

[pgen-0020086-b113] Boffelli D, Nobrega MA, Rubin EM (2004). Comparative genomics at the vertebrate extremes. Nat Rev Genet.

[pgen-0020086-b114] Dermitzakis ET, Reymond A, Lyle R, Scamuffa N, Ucla C (2002). Numerous potentially functional but non-genic conserved sequences on human chromosome 21. Nature.

[pgen-0020086-b115] Dermitzakis ET, Kirkness E, Schwarz S, Birney E, Reymond A (2004). Comparison of human chromosome 21 conserved nongenic sequences (CNGs) with the mouse and dog genomes shows that their selective constraint is independent of their genic environment. Genome Res.

[pgen-0020086-b116] Drake JA, Bird C, Nemesh J, Thomas DJ, Newton-Cheh C (2006). Conserved noncoding sequences are selectively constrained and not mutation cold spots. Nat Genet.

[pgen-0020086-b117] Gregory TR, Gregory TR (2005). Genome size evolution in animals. The evolution of the genome.

